# Preference for Stronger Taste Associated with a Higher Risk of Hypertension: Evidence from a Cross-Sectional Study in Northwest China

**DOI:** 10.1155/2022/6055940

**Published:** 2022-11-22

**Authors:** Huimeng Liu, Yutong Wang, Binyan Zhang, Yating Huo, Suixia Cao, Jingchun Liu, Lingxia Zeng, Hong Yan, Shaonong Dang, Baibing Mi

**Affiliations:** Department of Epidemiology and Biostatistics, School of Public Health, Xi'an Jiaotong University Health Science Center, Xi'an, Shaanxi 710061, China

## Abstract

**Background:**

Dietary modulation is a primary lifestyle approach for reducing the risk of hypertension. However, evidence of the potential role that a dietary taste preference plays in the risk of hypertension remains limited.

**Methods:**

A cross-sectional analysis was conducted based on the Shaanxi baseline survey of the Regional Ethnic Cohort Study. We used self-reported salt consumption and intensity preferences for sourness and spiciness to calculate the taste preference score, which was categorized into bland, moderate, and strong. A generalized linear mixed model and quantile regression were performed to estimate associations between taste preferences and hypertension/blood pressure.

**Results:**

Among 27,233 adults, 72.2% preferred a moderate taste and 21.4% preferred a strong taste. Compared with a bland taste, a stronger taste preference might be associated with a higher risk of hypertension (adjusted OR for a moderate taste = 1.25, 95% CI: 1.06, 1.49; adjusted OR for a strong taste = 1.41, 95% CI: 1.15, 1.71; *P*_trend_ = 0.002), especially in females (adjusted OR for a moderate taste = 1.43, 95% CI: 1.24, 1.66; adjusted OR for a strong taste = 1.55, 95% CI: 1.32, 1.83; *P*_trend_ < 0.001). Quantile regression showed that the taste preference was positively associated with diastolic blood pressure (DBP) (*P*_5_-*P*_80_) in females, with an average increase of 3.31 mmHg for a strong taste (*β* = 3.31, *P* < 0.001) and 1.77 mmHg for a moderate taste (*β* = 1.77, *P* = 0.008).

**Conclusions:**

A preference for stronger multitastes of salty, sour, and spicy might be associated with a higher risk of hypertension, especially in females. This relationship possibly occurs through increasing DBP. Dietary modulation with the promotion of a bland taste is encouraged.

## 1. Introduction

Hypertension, the leading modifiable risk factor for cardiovascular disease (CVD) and all-cause mortality, has become a major public health challenge worldwide over the past decades [1]. It was estimated to cause 8.5 million deaths worldwide in 2015 [2]. Approximately 1.28 billion people develop hypertension between 30 and 79 years, of which two-thirds come from low- and middle-income countries [3]. According to the China Hypertension Survey from 2012 to 2015, the standardized hypertension prevalence of Chinese adults was 23.2% (approximately 244.5 million) and another 41.3% (approximately 435.3 million) had prehypertension, which showed an increasing trend during the last twenty years [4]. In Northwest China, the estimated hypertension prevalence was 26.0%, which was higher than the prevalence in Southern China (20.0%) and slightly lower than the prevalence in Central China (27.3%) [5]. Regional heterogeneity in hypertension prevalence in China could be explained by variations in hypertension risk factors, such as obesity and an unhealthy diet [6]. It is essential to identify regional risk factors and take effective interventions to reduce the burden of hypertension, especially in developing countries [1].

Diet is one of the modifiable factors for reducing the risk of hypertension [7]. Taste preference, which refers to a composite sensation involving the perception of salty, sweet, sour, bitter, and umami, is a significant determinant of food selection and modulation [8] and can further influence body health. A data-based Internet study in China showed that dietary preference could reasonably predict the regional prevalence of diabetes, hypertension, and body mass index (BMI) [9]. Extensive research has explored the association between population health and single taste preferences. Substantial evidence has shown that high salt intake and a high salt taste recognition threshold increase the risk of coronary heart disease [10–13]. The hypertensive group benefited from decreasing salt intake more than the normotensive group in terms of a reduction in blood pressure (BP) [14]. Studies on spicy food found that spiciness played a beneficial role in cancer, obesity, and CVD, and the habitual consumption of spicy foods was inversely associated with mortality [15, 16]. Vinegar also protected health by attenuating postprandial glucose and insulin responses and reducing body weight [17, 18].

Taste preference was typically defined as a single pure taste in previous studies. However, in real-world situations, multiple tastes do not exist separately but rather in a composite and variant manner. A more accurate way of examining the influence of taste preferences is to model an indicator reflecting the composite sensation. Moreover, the dietary culture of regional taste preferences is developing and integrated, and it relates to local geographical (e.g., latitude, altitude, and climate) and economic situations [9]. The effect of taste preferences on health may be complicated and can be influenced by many factors, such as regional dietary habits, climate, and genetics [19, 20]. Shaanxi, located in Northwest China, with distinctive historical features and a combination of northern and southern cultures, has a unique pattern of taste preferences, but few studies have reported on it.

To fill these knowledge gaps, we aimed to build a taste preference score to evaluate a combined multitaste preference for salty, sour, and spicy and further explore the relationship between taste preferences and the risk of hypertension using the Shaanxi baseline dataset of the Regional Ethnic Cohort Study (RECS) in Northwest China.

## 2. Materials and Methods

### 2.1. Study Design and Participants

Detailed information about the design, implementation, and study population of RECS has been published elsewhere [21]. In this analysis, we obtained data from the baseline survey (conducted in 2018) of Shaanxi Province, one of the five Northwest regions included in the RECS. The baseline survey included a face-to-face questionnaire to review exposure risks and disease prevalence, biological specimen collection (including blood, saliva, and stool), and a regular medical examination. Participants with missing values for any taste preference of salty, sour, and spicy (*N* = 20,051), invalid reported daily salt consumption (extreme outliers of daily salt consumption were defined as higher than three times the interquartile range [22, 23], *N* = 321), missing (*N* = 22), and invalid (*N* = 2) values on outcomes were excluded from this study. In addition, 396 pregnant women were also excluded. The study flowchart is shown in Figure S1. Finally, 27,233 subjects aged 18–89 were included.

This study was conducted according to the guidelines in the Declaration of Helsinki, and all procedures involving research study participants were approved by the Human Research Ethics Committee of Xi'an Jiaotong University (No: XJTU2016-411). Written informed consent was obtained from all subjects.

### 2.2. Assessment of Hypertension

According to a standard protocol, BP measurement was conducted in a quiet room at a constant temperature, and BP was measured twice on the left arm in a sitting position at a 5-minute interval. If the difference between two measurements was more than 5 mmHg, a third measurement was conducted, and the average of the last two measurements was used for analysis. We also asked questions about formally diagnosed hypertension. According to the Chinese Guidelines for the Management of Hypertension, we defined hypertension as systolic blood pressure (SBP) greater than or equal to 140 mmHg and/or diastolic blood pressure (DBP) greater than or equal to 90 mmHg [24] or prediagnosed with hypertension.

### 2.3. Assessment of Taste Preferences

The taste preference score was calculated based on three main characteristics of dietary taste preferences in Shaanxi Province: salty, sour, and spicy [20]. In our study, we used the consumption of salt, vinegar, and spicy food to represent taste preferences for salty, sour, and spicy. In the questionnaire, we asked, “What strength of vinegar/spicy food do you usually prefer?,” “How many days is a bag of salt used?,” “What is the most common weight of a bag of salt?,” and “What is the total number of members in your family?.” We tested the reliability of the taste preference questionnaire with Cronbach's *α*, and the results (Cronbach's *α* = 0.636) indicated moderate internal consistency. The taste preference score calculation process details are presented in Supplementary Material 1 and Table S1. In short, we scored daily salt consumption per person according to quantiles and preferences for sour and spicy and then summed the scores of all three tastes. The total score ranged from 0 to 12, with a higher score representing a stronger taste preference. Finally, we trisected the total score and divided the taste preference into three groups: bland taste (taste score: 1-4), moderate taste (taste score: 5-8), and strong taste (taste score: 9-12).

### 2.4. Other Covariates

To facilitate choosing confounders for adjustment, we constructed a conceptual framework to visualize relationships among exposure, outcome, and confounders by using directed acyclic graphs (DAGs) (Figure S2) with the DAGitty program (https://dagitty.net/version2.3) [25, 26]. The minimal sufficient adjustment set comprised age (continuous), sex (female vs. male), educational attainment (middle school or below vs. above middle school), current drinking (no vs. yes), current smoking (no vs. yes), household income (<￥20,000, ￥20,000-￥100,000, and >￥100,000), meat intake frequency (seldom or never, 1–3 times per month, 1–3 times per week, 4–6 times per week, and every day), CVD history (no vs. yes), gastrointestinal disease history (no vs. yes), and mental disorder history (no vs. yes). Dietary information in the past year was collected through the Food Frequency Questionnaire (FFQ). Meat consumption included lamb, pork, and beef. CVD history was defined as the self-reported prevalence of stroke, angina, pulmonary heart disease, and rheumatic heart disease. Gastrointestinal disease history included gallstones, cirrhosis of the liver, and peptic ulcers. Mental disorder history included depression, excessive stress, self-inflicted faults, loss of interest, and reduced appetite in the past year. In addition, physical activity was determined by using a designed questionnaire that covered intensity, frequency, and time spent on four kinds of activities (occupational, commuting, household, and sports). Metabolic equivalents of task (METs) were estimated as the energy cost of a given activity divided by resting energy expenditure according to the Compendium of Physical Activities [27]. We multiplied the MET values for a particular type of physical activity by hours spent on that activity per day and summed MET hours for all activities to represent total physical activity in MET hours per day (MET-h/d).

Since diet assessment in our analysis was based on the FFQ without proportion data, it was difficult to calculate diet energy intake directly. We estimated daily energy based on the 2011 China Health and Nutrition Survey (CHNS) (*N* = 15,002) to resolve this limitation by the generalized linear model. The specific food items, model-building procedure, and results are shown in Supplementary Material 2 and Table S2.

### 2.5. Statistical Analysis

Descriptive statistics, including sociodemographic characteristics, dietary habits, health status, and lifestyle behaviour, are presented as a mean ± standard deviation (SD) for continuous variables or the number of participants (percentage) for categorical variables according to taste preference groups. ANOVA and chi-square tests were used to compare the distribution of the studied samples and the excluded samples and assess overall differences across taste preference groups.

In our study, by considering the group-specific effects of 7 study sites, we designed a generalized linear mixed model (GLIMMIX) with a random intercept (logit link function and binomial distribution) to estimate the contribution of taste preferences to hypertension. We applied taste preferences as a continuous variable (taste score) and a categorical variable (taste preference groups, using a bland taste as a reference) to the model. Multivariate models were adjusted for established and potential risk factors for hypertension. The crude model was built without covariate adjustments. The adjusted model was fully adjusted for age, sex, educational attainment, drinking status, smoking status, household income, meat intake frequency, CVD history, gastrointestinal disease history, mental disorder history, and estimated total energy intake. The interaction between preference for salty and spicy was estimated by adding a multiplicative term to the model. We further explored the association between spicy food preference and hypertension risk stratified by salt consumption groups. Quantile regression model was conducted with the same covariates in model 3 to compare the entiredistribution of BP between different taste preference groups. Furthermore, to explore the dose-response relationship between the taste score and the risk of hypertension, we selected the median imputed dataset and performed a restricted cubic spline (RCS) with 5 knots (5^th^, 10^th^, 50^th^, 90^th^, and 95^th^) to explore nonlinearity. Age was included in the regression model using an RCS function as a potential confounder. In addition, we also adjusted for the covariates mentioned above. The Wald test was used to test the nonlinearity of the observed relationship.

Subgroup analyses were conducted on social characteristics, lifestyles, and health status. The heterogeneity *P* value tested the significance of the interaction terms of subgroups and taste preference groups. Sensitivity analyses were performed to guarantee the robustness of the results. First, we reconducted analyses with self-reported hypertension and measured hypertension as a dependent variable to consider the change in dietary habits after hypertension. Second, to reduce selection bias due to missing data on confounders, we applied multivariate imputation (MI) by multivariate imputation by chained equations (MICEs) [28] under the assumption of missing at random (MAR). As suggested in literature [29], we included all relevant variables likely to be used in the subsequent analyses and generated five completed datasets.

Statistical analyses were performed with SAS version 9.4 (SAS Institute, Cary, NC, USA). All *P* values were two-sided, and we defined statistical significance as *P* < 0.05.

## 3. Results

### 3.1. Sample Characteristics

Among 27,233 studied participants, 6.4% preferred a bland taste, 72.2% preferred a moderate taste, and 21.4% preferred a strong taste. In total, 9,316 (34.21%) had self-reported or measured hypertension, of which 47.11% were already aware of their diagnosis. Comparisons between the studied and excluded samples are presented in Supplementary Material 3 and Table S3. The prevalence of hypertension among participants who preferred a bland, moderate, and strong taste was 29.30%, 34.08%, and 36.12%, respectively. Compared with bland tasters, strong tasters were more likely to be older, male, laborers, and smokers, with higher daily energy intake, higher BMI, higher DBP, and higher SBP, and more physical activity, but they were less likely to be educated or have a high household income (Table 1). Concerning the single taste preference distribution, we found that most people in Northwest China preferred eating sour and spicy food every day (81.30% preferred daily vinegar consumption, and 65.81% preferred spicy food every day) (Figure S3). Hypertension prevalence among taste preferences according to sex was presented in Figure S4, which showed higher prevalence among those who preferred a stronger taste, both for males and females.

### 3.2. Association between Taste Preferences and Hypertension

As shown in Table 2, the mean taste score was 7.11 ± 1.73 in all populations, and hypertension risk increased by 5% for every 1 point of increase (95% confidence interval (CI): 1.03, 1.06). The odds ratios (ORs) and 95% CIs for hypertension among strong tasters and moderate tasters were 1.40 (1.24, 1.59) and 1.29 (1.15, 1.44), respectively. In the fully adjusted model, the results showed that people who preferred a strong taste had a 41% higher risk (adjusted OR for a strong taste = 1.41, 95% CI: 1.15, 1.71) and those who preferred a moderate taste had a 25% higher risk (adjusted OR for a moderate taste = 1.25, 95% CI: 1.06, 1.49) of hypertension than people who preferred a bland taste (*P*_trend_ = 0.002). However, this trend was observed only in females, not in males. Among males (*P*_trend_ = 0.380), the adjusted OR (95% CI) of a strong taste was 0.96 (0.68, 1.35) and that of a moderate taste was 0.89 (0.66, 1.20). In females, the stronger their taste preference, the higher the risk of hypertension *P*_trend_ < 0.001. The adjusted OR (95% CI) of a strong taste was 1.55 (1.32, 1.83) and that of a moderate taste was 1.43 (1.24, 1.66). The association between a single taste preference and the risk of hypertension is presented in Table S4, and the results were similar to those of previous studies.

Considering that tastes may interact with each other, we further estimated the interaction effects of preference for salt and spicy tastes and conducted a stratified analysis (Table S5). The *P* value of the interaction was 0.019 in the fully adjusted model. After stratification by salt consumption groups, we found that a strong spicy preference combined with low salt consumption was not significantly associated with a risk of hypertension. For those with daily salt consumption higher than 3.33 g/day, the combination of strong spicy intake was associated with a higher risk of hypertension compared with no spicy intake, and the adjusted ORs and 95% CIs were 1.40 (1.12, 1.75) for the relatively low salt consumption group and 1.36 (1.07, 1.72) for the relatively high salt consumption group. However, we did not find a significant association between a strong spicy preference and hypertension risk in the high salt consumption group.

### 3.3. Association between Taste Preferences and BP

**Figure 1:**
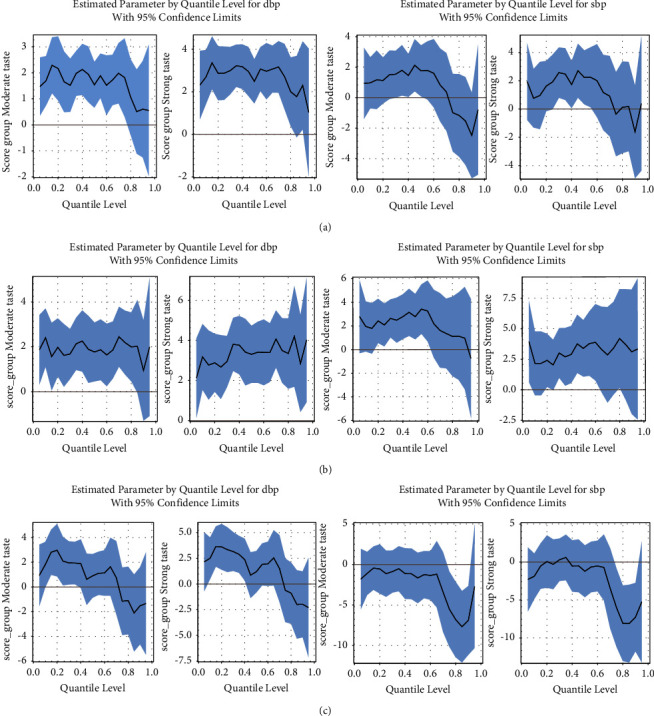
The estimated parameter at the quantile level of DBP (left) and SBP (right) of moderate taste and strong tastes with 95% CI in all populations and stratified by sex. (a) The estimated parameter at the quantile level of DBP and SBP of taste preference groups in all populations. (b) The estimated parameter at the quantile level of DBP and SBP of taste preference groups in females. (c) The estimated parameter at the quantile level of DBP and SBP of taste preference groups in males.

Figure 1 showed a more detailed quantile-related pattern of the association of SBP and DBP with taste preferences. Compared with the preference for a bland taste, a strong taste preference significantly increased DBP in 0.05∼0.85 percentiles (*β* = 2.00∼3.36 mmHg), with an average increase of 2.49 mmHg (*β* = 2.49, *P* < 0.001) (Figure 1(a)). However, no significant effect was observed on SBP (Figure 1(a)). In females, the taste preference was positively associated with DBP in nearly all percentiles, with an average increase of 3.31 mmHg for a strong taste (*β* = 3.31, *P* < 0.001) and 1.77 mmHg for a moderate taste (*β* = 1.77, *P* = 0.008) (Figure 1(b)). A positive association with SBP was found in *P*_5_, *P*_20_, and *P*_30_-*P*_80_ for a strong taste and *P*_20_‐*P*_60_ for a moderate taste (Figure 1(b)). However, for males, the taste preference was only positively associated with DBP in *P*_15_-*P*_35_ for a moderate taste and *P*_10_-*P*_40_ for a strong taste (Figure 1(c)). There was no significant association between taste preferences and SBP in all percentiles among males (Figure 1(c)).

### 3.4. Dose-Response Relationship between the Taste Score and Hypertension Risk

**Figure 2:**
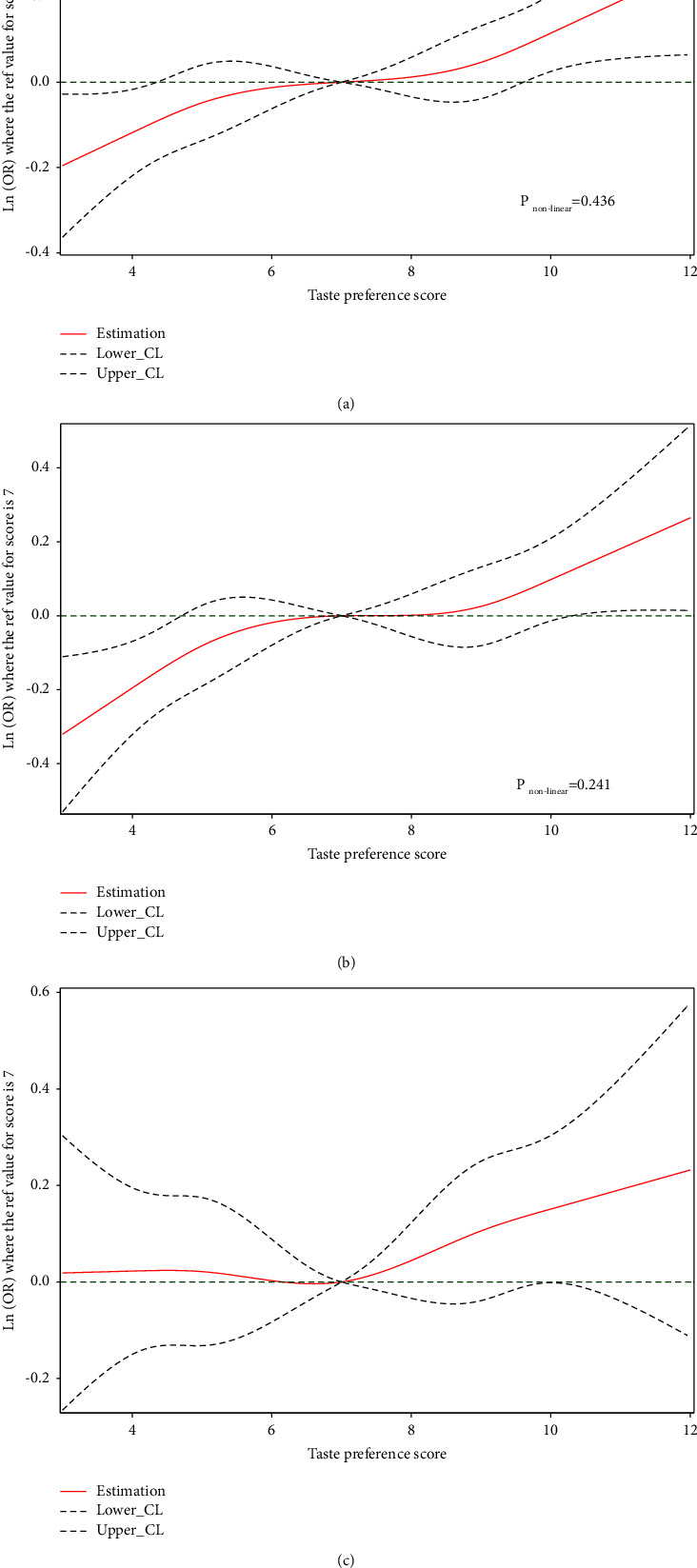
The restricted cubic spline for the taste preference score and hypertension risk, stratified by sex. Curves were fitted as a smooth term using a restricted cubic spline with 5 knots (5%, 25%, 50%, 75%, and 95%). The reference value was the median taste preference score. (a) The restricted cubic spline of the taste preference score and hypertension risk in all populations. (b) The restricted cubic spline of the taste preference score and hypertension risk in females. (c) The restricted cubic spline of the taste preference score and hypertension risk in males.

Figure 2 showed a dose-response relationship between the taste preference score and the risk of hypertension. In all populations, we observed a linear trend relationship between the taste score and hypertension risk (*P*_nonlinearity_ = 0.436). A bland taste (taste score <4) may well protect against hypertension, while a strong taste (taste score >10) may significantly increase hypertension risk (Figure 2(a)). For each additional point scored, hypertension risk may increase by 6% (adjusted OR = 1.06, 95% CI: 1.03–1.09) (Table 2). The results were almost identical for females (*P*_nonlinearity_ = 0.241) (Figure 2(b)). For each additional point scored, hypertension risk increased by 5% for females (adjusted OR = 1.05, 95% CI: 1.03–1.07). However, we observed no significant relationship in males (*P*_nonlinearity_ = 0.630).

### 3.5. Subgroup and Sensitivity Analysis

There were no significant differences between subgroups except for incomes and age groups. However, only the middle-income group showed a significant risk increase with a stronger taste preference (adjusted OR for a strong taste = 1.50, 95% CI: 1.14, 1.98; adjusted OR for a moderate taste = 1.49, 95% CI: 1.17, 1.90; *P*_heterogeneity_ = 0.044) (Figure S5). Among middle-aged participants, we observed that a strong taste preference was associated with a 42% higher risk of hypertension (adjusted OR = 1.42, 95% CI: 1.04, 1.94), but we did not observe a significant association between a moderate taste preference and the risk of hypertension.

Sensitivity analysis showed the robustness of the results. After changing the diagnostic definition of hypertension, the association between a one-point increase in the taste score and self-reported hypertension was slightly enhanced (adjusted OR for self-reported hypertension = 1.09, 95% CI: 1.06, 1.12; adjusted OR for measured hypertension = 1.05, 95% CI: 1.02, 1.08). Compared with a bland taste preference, a stronger taste preference was associated with a higher risk for self-reported and measured hypertension (self-reported hypertension: adjusted OR for a strong taste = 1.52, 95% CI: 1.22, 1.90; measured hypertension: adjusted OR for a strong taste = 1.31, 95% CI: 1.07, 1.60) (Table S6). The difference between sexes was similar to that in the previous results. A sensitivity analysis of imputed datasets also tested the robustness of our results (Table S7). A one-point increase in the taste score increased the risk of hypertension by 5% (adjusted OR = 1.05, 95% CI: 1.03, 1.06). Compared with a bland taste preference, a stronger taste preference was associated with a higher risk of hypertension in all populations (*P*_trend_ < 0.001). The adjusted ORs (95% CIs) were 1.30 (1.16, 1.470) and 1.42 (1.25, 1.62) for moderate and strong taste preferences, respectively. The results were consistent with the main findings after stratifying by sex, which showed that a stronger taste preference was positively associated with a higher risk of hypertension (*P*_trend_ for males = 0.276, *P*_trend_ for females <0.001).

## 4. Discussion

In this cross-sectional survey based on an RECS study with 27,233 participants in Shaanxi Province, we found that a stronger multitaste preference for salty, sour, and spicy was associated with a higher risk of hypertension, which may result from increased DBP, especially in females.

### 4.1. Comparison with Other Studies

Most recent studies have only explored the relationship between a single taste preference and hypertension risk. Considering the complexity of diet intake, we built a composite index to estimate a combined multitaste preference for salty, sour, and spicy. Moreover, we found interactive effects of salty and spicy tastes on the risk of hypertension. After stratification by salt consumption groups, people who preferred a strong spicy taste combined with low salt consumption showed a protective effect against hypertension (although not significant). In addition, a strong spicy taste preference was associated with a significantly higher risk of hypertension among those with daily salt consumption between 3.33 and 8.89 g/day. This result may indicate that excessive spicy food consumption in combination with salt may be positively associated with hypertension risk. However, no significant association was observed between a spicy taste preference and hypertension risk among people who preferred high salt intake (higher than 8.89 g/day). This may be because the strong association between salt consumption and the risk of hypertension may obscure the relationship between a spicy taste preference and hypertension risk. Several studies explored the interaction of salty and spicy taste preferences on BP, which was different from our findings. Using standard taste perception tests, a multicentre, random-order, double-blind observational study conducted in China found that enjoyment of spicy food reduced salt intake and BP by modifying the neural processing of salty tastes in the brain [30]. This inconsistency may be explained by regional differences in the environment and genetics. Climate, cooking methods, dietary habits, cultural customs, ethnic diversity, food production storage, and food transportation are critical environmental factors and may play a more dominant role in taste preferences [19]. Moreover, our descriptive analysis showed that the taste preference in Shaanxi Province was well known to be a strong preference for salty and spicy. Therefore, salty, sour, and spicy foods may synergistically affect hypertension.

The relationship between hypertension risk and taste preferences among females was robust even after imputing the missing data, which was in line with some other studies. A cross-sectional study using data from CHNS, including 9,273 participants, found that the frequency of spicy food consumption was inversely associated with the risk of hypertension in females but not in male adults [31]. Another cross-sectional study in Zhejiang Province, China, also found a significant negative relationship between the frequency of spicy food consumption and hypertension in females [32]. In our study, quantile-related patterns of taste preferences and BP were associated with DBP in nearly all percentiles among females. These results may suggest that females are more sensitive to the effect of taste preferences on DBP. Some studies emphasized that women potentially underwent several notable sex-specific changes that may impact their risk of hypertension [33]. For example, sex hormone changes can influence the vascular system, induce vasodilatation, inhibit vascular remodelling, and modulate the renin-angiotensin-aldosterone and sympathetic systems [34]. In our study, 85.46% of female participants were in menopause, when the risk of high BP doubles, even if corrected for other known CVD risk factors [35]. Furthermore, more studies with accurate taste preference measurements will be needed for further exploration.

### 4.2. Potential Biological Mechanism

Although the mechanism underlying the relationship between a strong taste preference and hypertension has not been fully elucidated, some possible hypotheses may be as follows: First, the transient receptor potential cation channel, subfamily V, member 1 (TRPV1), a TRP ion channel family member known as the capsaicin receptor, may contribute to the relationship between taste preferences and hypertension. TRPV1 has dual effects on different tissues [36]. Previous studies have found that spicy food consumption triggers sensory neuronal TRPV1, which releases several sensory neurotransmitters, including substance P and calcitonin gene-related peptide, resulting in vasodilation and depressurization [37]. However, vascular myocyte TRPV1 activation led to coronary constriction and a sustained increase in BP. In addition, the mediation of TRPV1 on BP regulation was independent of neurogenic regulation [38]. Second, high salt consumption was found to impair the expression of sensory neuronal TRPV1. An animal experiment showed that activating TRPV1 by capsaicin increased afferent renal nerve activity, but this increase was impaired in Dahl salt-sensitive rats with high salt intake. This impairment was not caused by elevated BP but may have resulted from salt loading [39].

### 4.3. Implications of the Findings

The taste preference score was built using the inspiration of the healthy lifestyle score, which was created by combining essential lifestyle factors relevant to the outcome based on prior knowledge [40]. Our study created a taste preference score by combining salt consumption with the degree of vinegar and spice consumption based on a real-world situation. The study results suggested that bland to moderate taste preferences might benefit females. This scoring method provided a simple way to assess the individual hypertension risk of taste preferences and provided further evidence supporting bland taste preferences for salty, sour, and spicy as potential strategies for hypertension prevention, especially in females.

### 4.4. Strengths and Limitations

The strengths of this study included the use of a large sample size of representative data from Northwest China, the use of standardized data collection procedures, and the consideration of multiple tastes. Several limitations merit mentioning when interpreting the results. First, the cross-sectional study restricted the establishment of causal relationships, and a reverse causal relationship might exist. Although we adjusted for multiple covariates, we could not control for some unmeasured confounders and residual covariates, which were also recognized as significant problems in cross-sectional studies. Second, generalization to other populations should be performed with caution because of the heterogeneity of the study samples and the excluded samples. In addition, the study was only conducted in Shaanxi Province, one of the northwest regions of China, and the results may not be suitable for other populations. Third, the taste preference for salty, sour, and spicy was self-reported, which might introduce recall bias, increase measurement error, and increase the risk of misclassification. More objective indicators of taste information will be needed in future studies. Fourth, although antihypertensive meditation is one of the crucial factors in hypertension research, we did not include it as a related factor in our study because of data unavailability, which may impact the estimation of the relationship. Finally, taste preferences change over time, and people may change their unhealthy habits after hypertension diagnosis. However, hypertensive patients also tended to report unhealthy lifestyles. These changes and recall bias may obscure the association between taste preferences and hypertension.

## 5. Conclusions

In conclusion, a stronger multitaste preference for sour, salty, and spicy foods might be associated with a higher risk of hypertension, especially in females. Moreover, the effects of taste preferences might affect DBP distribution. Our study emphasized the importance of bland tastes in hypertension prevention and indicated the complexity of the relationship between multiple tastes and hypertension risk. Thus, future studies need to apply data from different studies (such as clinical trials and animal trials), collect authentic taste information, and consider the environmental and genetic influence on individual taste preferences.

## Figures and Tables

**Table 1:** Baseline characteristics of the study population according to taste groups^a^.

Characteristics	Taste group			*P* value
	Bland taste (taste score <4, *N* = 1,751)	Moderate taste (taste score between 5 to 8, *N* = 19,655)	Strong taste (taste score between 9 to 12, *N* = 5,827)	
Mean age (years)	52.78 ± 12.93	54.63 ± 10.36	55.71 ± 8.82	<0.001
Sex				
Male (%)	565 (32.27)	6364 (32.38)	2063 (35.40)	<0.001
Female (%)	1186 (67.73)	13291 (67.62)	3764 (64.60)	
Race				
Han (%)	1736 (99.20)	19501 (99.51)	5786 (99.47)	0.221
Other ethnic (%)^b^	14 (0.80)	96 (0.49)	31 (0.53)	
Educational attainment				
Middle school or below (%)	1198 (68.57)	15592 (79.39)	5065 (86.97)	<0.001
Above middle school (%)	549 (31.43)	4047 (20.61)	759 (13.03)	
Occupation				
Laborer (%)	550 (31.72)	9825 (50.34)	3807 (65.83)	
Housewife (%)	576 (33.22)	5757 (29.49)	1366 (23.62)	<0.001
Worker (%)	115 (6.63)	906 (4.64)	148 (2.56)	
Business (%)	40 (2.31)	277 (1.42)	71 (1.23)	
Executives (%)	137 (7.90)	673 (3.45)	57 (0.99)	
Unemployed (%)	29 (1.67)	233 (1.19)	39 (0.67)	
Professional and technical personnel (%)	166 (9.57)	900 (4.61)	109 (1.88)	
Sales man (%)	69 (3.98)	643 (3.29)	112 (1.94)	
Others (%)	52 (3.00)	305 (1.56)	74 (1.28)	
Household income (yuan/year)				
<20000 (%)	516 (31.87)	5801 (30.80)	1714 (30.18)	<0.001
20000–100000 (%)	913 (56.39)	11975 (63.58)	3831 (67.45)	
>100000 (%)	190 (11.74)	1059 (5.62)	135 (2.38)	
Current smoking (%)	267 (15.40)	3304 (16.88)	1285 (22.13)	<0.001
Current drinking (%)	451 (25.85)	3953 (20.15)	1122 (19.27)	<0.001
Energy intake (kcal/d)	1668.37 ± 246.67	1742.75 ± 106.75	1775.07 ± 197.36	<0.001
Daily salt consumption (g/d)	2.64 ± 1.34	6.16 ± 3.96	10.74 ± 4.29	<0.001
Meat intake frequency				
Every day	313 (18.02)	3701 (18.87)	1151 (19.80)	<0.001
4–6 times per week	341 (19.63)	1990 (10.15)	510 (8.77)	
1–3 times per week	549 (31.61)	4661 (23.77)	1147 (19.73)	
1–3 times per month	283 (16.29)	4844 (24.70)	1403 (24.13)	
Seldom or never	251 (14.45)	4414 (22.51)	1603 (27.57)	
Physical activity (MET/day)^c^	20.72 ± 18.03	23006 ± 17.28	24.59 ± 17.44	<0.001
BMI	23.67 ± 3.34	23.84 ± 3.38	24.40 ± 4.24	<0.001
Underweight (%)	101 (5.90)	903 (4.67)	224 (3.91)	
Normal weight (%)	911 (53.21)	9444 (48.88)	2567 (44.81)	
Overweight (%)	544 (31.78)	6911 (35.77)	2146 (37.46)	<0.001
Obesity (%)	156 (9.11)	2064 (10.68)	792 (13.82)	
Anthropometric measurements				
Height (cm)	158.78 ± 12.93	159.14 ± 8.49	159.54 ± 8.03	<0.001
Weight (kg)	59.28 ± 11.37	60.53 ± 10.63	61.87 ± 10.59	<0.001
Waist (cm)	81.19 ± 9.58	82.23 ± 9.55	82.81 ± 9.78	<0.001
SBP (mmHg)	125.37 ± 20.09	125.62 ± 18.98	125.82 ± 18.72	0.648
DBP (mmHg)	79.01 ± 11.48	80.84 ± 11.05	81.62 ± 10.86	<0.001
Measured hypertension (%)	449 (25.75)	5524 (28.19)	1701 (29.23)	0.017
Hypertension awareness (%)^d^	191 (11.24)	3017 (16.01)	1028 (18.46)	<0.001
Hypertension (%)^e^	513 (29.3)	6698 (34.08)	2105 (36.12)	<0.002
Health status				
Had CVD disorder history (%)	140 (8.27)	1677 (9.03)	570 (1037)	0.004
Had gastrointestinal disease history (%)	20.72 ± 18.03	23006 ± 17.28	24.59 ± 17.44	<0.001
Mental disorder history (%)	1725 (99.60)	19473 (99.47)	5787 (99.47)	0.777

^a^For some variables, the sum of categories is not equal to the total due to missing data. ^b^Other ethnicities include the Uygur, Kazakh, Hui, Mongolian, and Tibetan ethnic groups. ^c^Physical activity was calculated by multiplying the metabolic equivalent task (MET) value for a particular type of physical activity by the hours spent on that activity per day and summing the MET-hours for all activities. ^d^Hypertension awareness was defined according to self-reported hypertension. ^e^Hypertension was defined by either measured or self-reported hypertension. MET metabolic equivalent of task; BMI body mass index, CVD cardiovascular disease; SBP systolic blood pressure; DBP diastolic blood pressure.

**Table 2:** Odds ratios (95% CIs) of hypertension across taste scores for all participants.

Variables	Mean (SD)/case (%)	Crude model^a^	*P* value^b^	Adjusted model^c^	*P* value^b^

All population					
Taste score (per increase for 1 point)	7.11 (1.73)	1.05 (1.03,1.06)	<0.001	1.06 (1.03,1.09)	<0.001
Taste group					0.002
Bland taste	513 (29.30)	Reference	<0.001	Reference	
Moderate taste	6698 (34.08)	1.29 (1.15,1.44)		1.25 (1.06,1.49)	
Strong taste	2105 (36.12)	1.40 (1.24,1.59)		1.41 (1.15,1.71)	

Male participants					
Taste score (per increase for 1 point)	7.08 (1.73)	1.03 (1.00,1.05)	0.063	1.02 (0.97,1.07)	0.380
Taste group					
Bland taste	191 (33.82)	Reference	0.129	Reference	0.805
Moderate taste	2336 (36.71)	1.08 (0.90,1.31)		0.89 (0.66,1.20)	
Strong taste	792 (38.39)	1.16 (0.94,1.43)		0.96 (0.68,1.35)	

Female participants					
Taste score (per increase for 1 point)	7.17 (1.74)	**1.05 (1.03,1.07)**	**<0.001**	**1.05 (1.03,1.07)**	**<0.001**
Taste group					
Bland taste	322 (27.15)	Reference	**<0.001**	Reference	**<0.001**
Moderate taste	4362 (32.82)	**1.39 (1.21,1.60)**		**1.43 (1.24,1.66)**	
Strong taste	1313 (34.88)	**1.51 (1.30,1.76)**		**1.55 (1.32,1.83)**	

^a^Crude model without covariate adjustments. ^b^For categorical taste preference groups, the *P* value represents trend *P*.^c^Fully adjusted for age, sex, educational attainment, current drinking, current smoking, household income, meat intake frequency, cardiovascular disease history, gastrointestinal disease history, mental disorder history, chronic kidney disease history, and estimated total energy intake.

## Data Availability

The datasets used and/or analyzed during the current study are available from the corresponding author upon reasonable request.

## Supplementary Materials

Supplementary Materials Supplementary Materials were included as a separate file.
