# Supplementary material for: Preference for Stronger Taste Associated with a Higher Risk of Hypertension: Evidence from a Cross-Sectional Study in Northwest China
Source: Int J Hypertens. 2022 Nov 22;2022:6055940. doi: 10.1155/2022/6055940 (PMC9708377; doi:10.1155/2022/6055940)
Supplement: Supplementary Materials — were included as a separate file. [file 6055940.f1.docx]

**Supplementary Materials**

**Preference for Stronger Taste Associated with a Higher Risk of Hypertension: Evidence from a Cross-sectional Study in Northwest China**

**Supplementary Material 1: Calculation of taste preference score**

Based on the questionnaire, we calculated the daily salt consumption per person. Daily salt consumption higher than the 75^th^ percentile was assigned a score of 4, and other groups scored miner 1 in order. 4, 3, 2, and 1 were assigned for strong, moderate, weak, and no spicy food or vinegar consumption, respectively (**Table S1**). We summed the score of every single taste, ranging from 0 to 12, and a higher score meant a stronger taste. Finally, we trisected the total score and divided the taste preference into three groups: bland taste (taste score: 1-4), moderate taste (taste score: 5-8), and strong taste (taste score: 9-12).

Table S1. Taste preference component and scoring

| **Taste preference** | **Category standards** | **Weight (scores)** |
| --- | --- | --- |
| Salty | Salt consumption>=8.89 g/day | 4 |
|  | 6.15<=Salt consumption<8.89 g/day | 3 |
|  | 3.33<=Salt consumption<6.15 g/day | 2 |
|  | Salt consumption<3.33 g/day | 1 |
| Sour | Favorite, high volume | 4 |
|  | Like, moderate volume | 3 |
|  | Moderate, low volume | 2 |
|  | Dislike, does not eat | 1 |
| Spicy | Strong spicy | 4 |
|  | Moderate spicy | 3 |
|  | Weak spicy | 2 |
|  | Not spicy | 1 |

**Supplementary Material 2: Daily energy estimation based on China Health and Nutrition Survey (CHNS)**

To minimize energy calculation limitations, we built daily energy estimated linear model based on the China Health and Nutrition Survey (CHNS), including 13 foods (**Table S2**). We used the CHNS data set (N=15,002) conducted in 2011, which was close to our survey. Daily total energy intake in CHNS was calculated based on individual food composition data using the Chinese Food Composition Table. In our study, we first transform times of having specific food in the past 3 days into food consumption frequency. For example, if they reported having rice 3 times in the past 3 days, we defined it as eating rice every day; if they reported 2 times, we defined it as 4-6 times per week. Finally, the frequency of eating specific food was categorized into four groups: every day, 4-6 times per week, 1-3 times per week, seldom, or never. We then built a Generalized Linear Model with daily energy intake as the dependent variable and frequency of 13 food items as independent variables. Modeling results are presented in **Table S2**.

Table S2. Parameter estimate of daily energy estimate model based on CHNS 2011

| **Variable** | **Estimate** | **Standard derivation** | ***t* value** | ***P* value** |
| --- | --- | --- | --- | --- |
| Intercept | 2633.33 | 243.57 | 10.81 | <0.001 |
| Frequency of rice consumption | -4.87 | 16.98 | -0.29 | 0.774 |
| Frequency of noodle consumption | -71.15 | 12.04 | -5.91 | <0.001 |
| Frequency of grain consumption | -34.56 | 14.54 | -2.38 | 0.018 |
| Frequency of port consumption | -89.44 | 12.47 | -7.17 | <0.001 |
| Frequency of lamp consumption | 28.51 | 51.93 | 0.55 | 0.583 |
| Frequency of beef consumption | -17.27 | 23.87 | -0.72 | 0.469 |
| Frequency of chicken consumption | -38.49 | 18.11 | -2.13 | 0.034 |
| Frequency of seafood consumption | -4.74 | 15.26 | -0.31 | 0.756 |
| Frequency of egg consumption | -17.37 | 12.48 | -1.39 | 0.164 |
| Frequency of vegetable consumption | -89.11 | 33.21 | -2.68 | 0.007 |
| Frequency of fruit consumption | -4.22 | 10.65 | -0.40 | 0.692 |
| Frequency of tomatoes consumption | 30.65 | 13.61 | 2.25 | 0.024 |
| Frequency of beans consumption | -36.36 | 13.07 | -2.78 | 0.005 |

**Supplementary Material 3: Comparison between study samples and excluded samples**

Compared with the excluded samples, the samples in this study included older participants (mean age: 54.73±10.26), fewer males (8,992 [33.02%]), less educated (16,669 [50.85%]) participants, more laborers (14,182 [52.46%]), participants with lower household income (30.73% of participants’ income less than 20,000 per year). Those participants seem to less smoking (4,856 [17.91%]), drinking (5,526 [20.33%]), daily energy intake (1745.33±208.72), and physical activity (mean MET/day: 21.79±15.99) with higher BMI (23.92±3.59) and blood pressure (mean SBP: 125.65±19.00, mean DBP: 80.89±11.05) (**Table S3**).

Table S3. Baseline characteristic comparison between the studied and excluded samples

| **Characteristics** | **Study sample (N=27,233)** | **Excluded sample**  **(N=19,164)** | | ***P* value** |
| --- | --- | --- | --- | --- |
| Age | 54.73±10.26 | 42.91±13.95 | | <0.001 |
| Male (%) | 8992 (33.02) | 9521 (49.68) | | <0.001 |
| **Race** |  |  | |  |
| Han (%) | 27023 (99.48) | 18775 (98.58) | | <0.001 |
| Other Ethnicity (%)^a^ | 141 (0.52) | 270 (1.42) | |  |
| **Education attainment** |  |  | |  |
| Middle school or below (%) | 16669 (50.85) | 9129 (67.42) | | <0.001 |
| Above middle school (%) | 16121 (49.16) | 1047 (32.58) | |  |
| **Occupation** |  |  | |  |
| Laborer (%) | 14,182 (52.46) | 1745 (9.45) | | <0.001 |
| Housewife (%) | 7699 (28.48) | 1828 (9.90) | |  |
| Worker (%) | 1169 (4.32) | 2369 (12.84) | |  |
| Business (%) | 388 (1.44) | 180 (0.98) | |  |
| Executive (%) | 867 (3.21) | 5310 (28.77) | |  |
| Unemployed (%) | 301 (1.11) | 125 (0.68) | |  |
| Professional and technical personnel | 1175 (4.35) | 3829 (20.75) | |  |
| Salesperson (%) | 824 (3.05) | 1436 (7.78) | |  |
| Others (%) | 431 (1.59) | 1634 (8.85) | |  |
| **Household income (yuan/year)** |  |  | |  |
| <20,000 (%) | 8031 (30.73) | 2881 (19.51) | | <0.001 |
| 20,000-100,000 (%) | 16719 (63.97) | 6881 (46.60) | |  |
| >100,000 (%) | 1384 (5.30) | 5003 (33.88) | |  |
| **Current smoking** | 4856 (17.91) | 4775 (25.60) | | <0.001 |
| **Current drinking** | 5526 (20.33) | 10377 (55.10) | | <0.001 |
| **Energy intake (kcal/d)** | 1745.33±208.72 | 1780.67±229.43 | | <0.001 |
| **Daily salt consumption (g/d)** | 6.91±4.48 | 14.50±18.41 | | <0.001 |
| **Meat intake frequency** |  |  | |  |
| Every day (%) | 5165 (19.02) | 4194 (23.19) | | <0.001 |
| 4-6 times per week (%) | 2841 (10.46) | 3795 (20.98) | |  |
| 1-3 times per week (%) | 6357 (23.40) | 6015 (33.25) | |  |
| 1-3 times per month (%) | 6530 (24.04) | 2470 (13.66) | |  |
| Seldom or never | 6268 (23.08) | 1614 (8.92) | |  |
| **Physical Activity (MET/day)^b^** | 21.79±15.99 | 25.43±16.87 | | <0.001 |
| **BMI** | 23.92±3.59 | 23.72±3.45 | | <0.001 |
| Underweight (%) | 1228 (4.59) | 1308 (6.93) | | <0.001 |
| Normal weight (%) | 12,922 (48.28) | | 9376 (49.71) |  |
| Overweight (%) | 9601 (35.87) | 6187 (32.80) | |  |
| Obese (%) | 3012 (11.25) | 1992 (10.56) | |  |
| **Anthropometric-measurements** |  |  | |  |
| Height (cm) | 159.18±8.46 | 166.33±115.57 | | <0.001 |
| Weight (kg) | 60.74±10.69 | 65.27±12.82 | | <0.001 |
| Waist (cm) | 82.30±9.61 | 81.86±12.72 | | <0.001 |
| **Blood Pressure** |  |  | |  |
| SBP | 125.65±19.00 | 120.20±16.49 | | <0.001 |
| DBP | 80.89±11.05 | 78.16±11.06 | | <0.001 |
| Measured hypertension (%) | 7674 (28.25) | 3446 (18.35) | | <0.001 |
| Hypertension awareness (%)^c^ | 4236 (16.22) | 1809 (10.47) | | <0.001 |
| Hypertension (%)^d^ | 9316 (34.21) | 4225 (22.12) | | <0.001 |
| **Health status** |  |  | |  |
| Had CVD disorder history (%) | 2387 (9.26) | 897 (5.24) | | <0.001 |
| Had gastrointestinal disease history (%) | 1358 (12.99) | 1269 (24.59) | | <0.001 |
| Mental disorder history (%) | 26985 (99.48) | 17083 (98.14) | | <0.001 |

a.Other Ethnicities include the Uygur, Kazakh, Hui, Mongolian, and Tibetan ethnic groups

b. Physical activity was calculated by multiplying the metabolic equivalent tasks (METs) value for a particular type of physical activity by the hours spent on that activity per day and summing the MET-hours for all activities.

c. Hypertension awareness was defined according to self-reported hypertension.

d. Hypertension was defined by either the measured or self-reported hypertension.

Table S4. Association of single taste preference and hypertension

| Variable | Case (%) | Model 1 | *P*_trend_ | Model 2 | *P*_trend_ | Model 3 | *P*_trend_ |
| --- | --- | --- | --- | --- | --- | --- | --- |
| **Salt consumption group** |  |  |  |  |  |  |  |
| Salt consumption>=8.89 g/day | 2396 (35.33) | 1.07 (0.98,1.16) | 0.3874 | **1.16 (1.01,1.34)** | 0.9253 | 1.10 (0.95,1.27) | 0.8831 |
| 6.15<=Salt consumption<8.89 g/day | 2387 (34.32) | 1.02 (0.94,1.11) |  | **1.16 (1.01,1.34)** |  | 1.12 (0.97,1.29) |  |
| 3.33<=Salt consumption<6.15 g/day | 2905 (35.28) | 1.07 (0.99,1.15) |  | **1.25 (1.09,1.44)** |  | **1.23 (1.07,1.42)** |  |
| Salt consumption<3.33 g/day | 1628 (30.93) | Reference |  | Reference |  | Reference |  |
| **Sour preference** |  |  |  |  |  |  |  |
| Favorite, high volume | 1188 (38.42) | **1.70 (1.52,1.91)** | **<0.001** | **1.69 (1.40,2.05)** |  | **1.62 (1.34,1.97)** | **<0.001** |
| Like, moderate volume | 3541 (33.55) | **1.42 (1.29,1.57)** |  | **1.59 (1.36,1.87)** |  | **1.54 (1.30,1.81)** |  |
| Moderate, low volume | 3712 (34.47) | **1.37 (1.24,1.50)** |  | **1.28 (1.10,1.48)** | **<0.001** | **1.24 (1.06,1.44)** |  |
| Dislike, does not eat | 875 (31.07) | Reference |  | Reference |  | Reference |  |
| **Spicy preference** |  |  |  |  |  |  |  |
| Strong spicy | 6397 (35.87) | 0.91 (0.78,1.06) | **<0.001** | 1.06 (0.82,1.36) | **0.026** | 0.94 (0.73,1.21) | 0.140 |
| Moderate spicy | 314 (27.05) | **1.09 (1.01,1.18)** |  | **1.23 (1.09,1.39)** |  | 1.10 (0.97,1.24) |  |
| Weak spicy | 1372 (29.67) | 0.94 (0.85,1.04) |  | 1.09 (0.94,1.27) |  | 1.06 (0.91,1.23) |  |
| Not spicy | 1181 (33.95) | Reference |  | Reference |  | Reference |  |

Model 1: crude model without covariates adjustment

Model2: fully adjusted for age, sex, education attainment, current drinking, current smoking, household income, meat intake frequency, daily salt consumption per person, cardiovascular disease history, gastrointestinal disease history, mental disorder history, chronic kidney disease history, and estimated total energy intake.

Model3: fully adjusted for age, sex, education attainment, current drinking, current smoking, household income, meat intake frequency, daily salt consumption per person, cardiovascular disease history, gastrointestinal disease history, mental disorder history, chronic kidney disease history, estimated total energy intake, and additionally other two tastes preferences.

Table S5 The association between spicy preference and hypertension stratified by salt preference

| Spicy preference | Case (%)^a^ | Crude model^b^ | *P* value | Adjusted model^c^ | *P* value |
| --- | --- | --- | --- | --- | --- |
| **Low salt (salt consumption<3.33 g/day)** | | | | | |
| No spicy | 702 (12.82) | Reference |  | Reference |  |
| Weak spicy | 1679 (30.67) | 0.84 (0.69,1.03) | 0.101 | 1.03 (0.72,1.48) | 0.855 |
| Moderate spicy | 467 (8.53) | **0.73 (0.54,0.99)** | **0.045** | 0.82 (0.46,1.47) | 0.506 |
| Strong spicy | 2626 (47.97) | 0.96 (0.80,1.15) | 0.653 | 0.82 (0.60,1.12) | 0.202 |
| **Relatively low salt (3.33<=Salt consumption<6.15 g/day)** | | | | | |
| No spicy | 1094 (13.01) | Reference |  | Reference |  |
| Weak spicy | 1448 (17.22) | 0.94 (0.79,1.12) | 0.491 | 0.97 (0.74,1.27) | 0.801 |
| Moderate spicy | 393 (4.67) | 0.93 (0.71,1.22) | 0.602 | 1.06 (0.68,1.63) | 0.809 |
| Strong spicy | 5474 (65.1) | **1.27 (1.10,1.46)** | **0.001** | **1.40 (1.12,1.75)** | **0.003** |
| **Relatively high salt (6.15<=Salt consumption<8.89 g/day)** | | | | | |
| No spicy | 882 (12.56) | Reference |  | Reference |  |
| Weak spicy | 904 (12.88) | 1.07 (0.87,1.31) | 0.519 | 1.28 (0.95,1.72) | 0.106 |
| Moderate spicy | 219 (3.12) | 1.18 (0.85,1.65) | 0.329 | 1.20 (0.73,1.98) | 0.471 |
| Strong spicy | 5016 (71.44) | **1.18 (1.00,1.38)** | **0.046** | **1.36 (1.07,1.72)** | **0.011** |
| **Strong salt (Salt consumption>=8.89 g/day)** | | | | | |
| No spicy | 900 (12.58) | Reference |  | Reference |  |
| Weak spicy | 865 (12.09) | 0.91 (0.74,1.11) | 0.334 | 1.03 (0.76,1.39) | 0.851 |
| Moderate spicy | 193 (2.7) | 0.86 (0.61,1.21) | 0.388 | 0.94 (0.54,1.62) | 0.818 |
| Strong spicy | 5194 (72.62) | 0.95 (0.81,1.11) | 0.485 | 1.04 (0.82,1.31) | 0.760 |

^a^ Do not match the total number because of missing data.

^b^ Crude model without covariates adjustment.

^c^ Fully adjusted for age, sex, education attainment, current drinking, current smoking, household income, meat intake frequency, daily salt consumption per person, cardiovascular disease history, gastrointestinal disease history, mental disorder history, chronic kidney disease history, and estimated total energy intake.

Table S6. Odds ratios (95% CI) of taste preference associated with self-reported hypertension or measured hypertension

| **Variable** | **Mean (SD)/Case (%)** | **Crude model^a^** | ***P* value^b^** | **Adjusted model^c^** | ***P* value^b^** |
| --- | --- | --- | --- | --- | --- |
| **Self-reported hypertension** | | | | | |
| **All populations** |  |  |  |  |  |
| Taste Score (per increase for 1 point) | 7.11 (1.74) | **1.06 (1.04,1.08)** | **<0.001** | **1.09 (1.06,1.12)** | **<0.001** |
| Taste Group |  |  |  |  |  |
| Bland taste | 1028 (18.46) | Reference | **<0.001** | Reference | **<0.001** |
| Moderate taste | 3017 (16.01) | **1.28 (1.09,1.50)** |  | **1.35 (1.11,1.66)** |  |
| Strong taste | 191 (11.24) | **1.40 (1.18,1.66)** |  | **1.52 (1.22,1.90)** |  |
| **Male participants** |  |  |  |  |  |
| Taste Score (per increase for 1 point) | 7.17 (1.74) | 1.03 (1.00,1.07) | 0.089 | 1.03 (0.98,1.09) | 0.217 |
| Taste Group |  |  |  |  |  |
| Bland taste | 64 (11.66) | Reference | 0.264 | Reference | 0.727 |
| Moderate taste | 1022 (16.81) | 1.29 (0.97,1.69) |  | 1.25 (0.87,1.78) |  |
| Strong taste | 356 (18.10) | 1.30 (0.97,1.76) |  | 1.20 (0.81,1.78) |  |
| **Female participants** |  |  |  |  |  |
| Taste Score (per increase for 1 point) | 7.08 (1.73) | **1.07 (1.04,1.10)** | **<0.001** | **1.11 (1.07,1.15)** | **<0.001** |
| Taste Group |  |  |  |  |  |
| Bland taste | 127 (11.03) | Reference | **<0.001** | Reference | **<0.001** |
| Moderate taste | 1995 (15.63) | **1.26 (1.04,1.53)** |  | **1.45 (1.13,1.86)** |  |
| Strong taste | 672 (18.66) | **1.44 (1.16,1.77)** |  | **1.77 (1.34,2.33)** |  |
| **Measured hypertension** | | | | | |
| **All populations** |  |  |  |  |  |
| Taste Score (per increase for 1 point) | 7.11 (1.74) | **1.04 (1.03,1.06)** | **<0.001** | **1.05 (1.02,1.08)** | **<0.001** |
| Taste Group |  |  |  |  |  |
| Bland taste | 1701 (29.23) | Reference | **<0.001** | Reference | **0.007** |
| Moderate taste | 5524 (28.19) | **1.27 (1.13,1.43)** |  | 1.16 (0.97,1.39) |  |
| Strong taste | 449 (25.75) | **1.38 (1.22,1.57)** |  | **1.31 (1.07,1.60)** |  |
| **Male participants** |  |  |  |  |  |
| Taste Score (per increase for 1 point) | 7.17 (1.74) | **1.03 (1.00,1.06)** | 0.061 | 1.03 (0.98,1.08) | 0.270 |
| Taste Group |  |  |  |  |  |
| Bland taste | 167 (29.72) | Reference | 0.126 | Reference | 0.506 |
| Moderate taste | 1947 (30.69) | 1.09 (0.89,1.32) |  | 0.90 (0.66,1.22) |  |
| Strong taste | 648 (31.43) | 1.16 (0.94,1.45) |  | 1.01 (0.72,1.42) |  |
| **Female participants** |  |  |  |  |  |
| Taste Score (per increase for 1 point) | 7.08 (1.73) | **1.05 (1.02,1.07)** | **<0.001** | **1.06 (1.03,1.10)** | **<0.001** |
| Taste Group |  |  |  |  |  |
| Bland taste | 282 (23.86) | Reference | **<0.001** | Reference | **0.005** |
| Moderate taste | 3577 (26.99) | **1.37 (1.19,1.58)** |  | **1.26 (1.02,1.57)** |  |
| Strong taste | 1053 (28.02) | **1.48 (1.26,1.74)** |  | **1.43 (1.11,1.84)** |  |

^a^ Crude model without covariates adjustment.

^b^ For categorical taste preference groups, *P* value represent trend *P.*

^c^ Fully adjusted for age, sex, education attainment, current drinking, current smoking, household income, meat intake frequency, daily salt consumption per person, cardiovascular disease history, gastrointestinal disease history, mental disorder history, chronic kidney disease history, and estimated total energy intake.

**Abbreviations SD s**tandard deviation, **CVD** cardiovascular disease

Table S7. Odds ratios (95% CI) of taste preference associated with hypertension after multiple imputations

| **Variable** | **Crude model^a^** | ***P* value^b^** | **Adjusted model^c^** | ***P* value^b^** |
| --- | --- | --- | --- | --- |
| **All population** |  |  |  |  |
| Taste Score (per increase for 1 point) | **1.05 (1.03,1.06)** | **<0.001** | **1.05 (1.03,1.06)** | **<0.001** |
| Taste Group |  |  |  |  |
| Bland taste | Reference | **<0.001** | Reference | **<0.001** |
| Moderate taste | **1.29 (1.15,1.44)** |  | **1.30 (1.16,1.47)** |  |
| Strong taste | **1.40 (1.24,1.59)** |  | **1.42 (1.25,1.62)** |  |
| **Male participants** |  |  |  |  |
| Taste Score (per increase for 1 point) | **1.03 (1.00,1.05)** | 0.063 | **1.03 (1.00,1.06)** | **0.048** |
| Taste Group |  |  |  |  |
| Bland taste | Reference |  | Reference |  |
| Moderate taste | 1.08 (0.90,1.31) | 0.129 | 1.02 (0.83,1.26) | 0.276 |
| Strong taste | 1.16 (0.94,1.43) |  | 1.09 (0.86,1.39) |  |
| **Female participants** |  |  |  |  |
| Taste Score (per increase for 1 point) | **1.05 (1.03,1.07)** | **<0.001** | **1.09 (1.06,1.12)** | **<0.001** |
| Taste Group |  |  |  |  |
| Bland taste | Reference | **<0.001** | Reference | **<0.001** |
| Moderate taste | **1.39 (1.21,1.60)** |  | **1.57 (1.35,1.83)** |  |
| Strong taste | **1.51 (1.30,1.76)** |  | **1.85 (1.55,2.20)** |  |

^a^ Crude model without covariates adjustment.

^b^ For categorical taste preference groups, *P* value represent trend *P*

^c^ Fully adjusted for age, sex, education attainment, current drinking, current smoking, household income, meat intake frequency, daily salt consumption per person, cardiovascular disease history, gastrointestinal disease history, mental disorder history, chronic kidney disease history, and estimated total energy intake.

**Supplementary Figures**


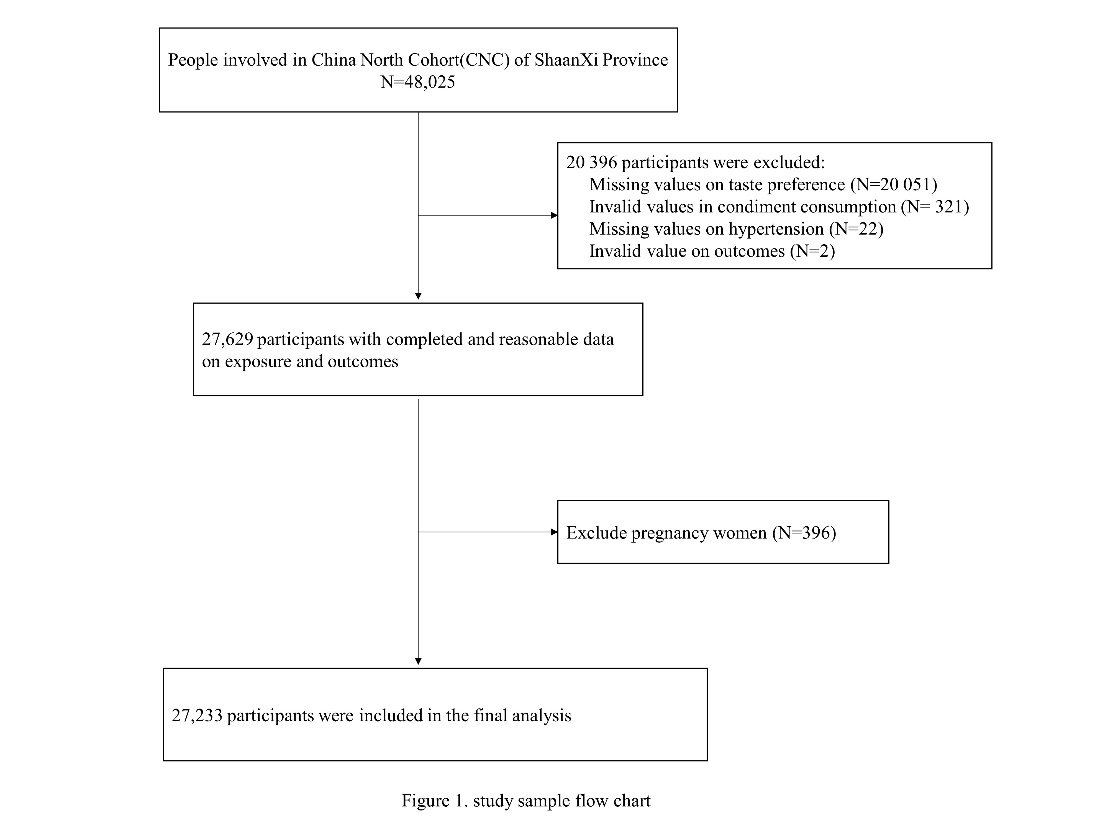


Figure S1: Study flow chart


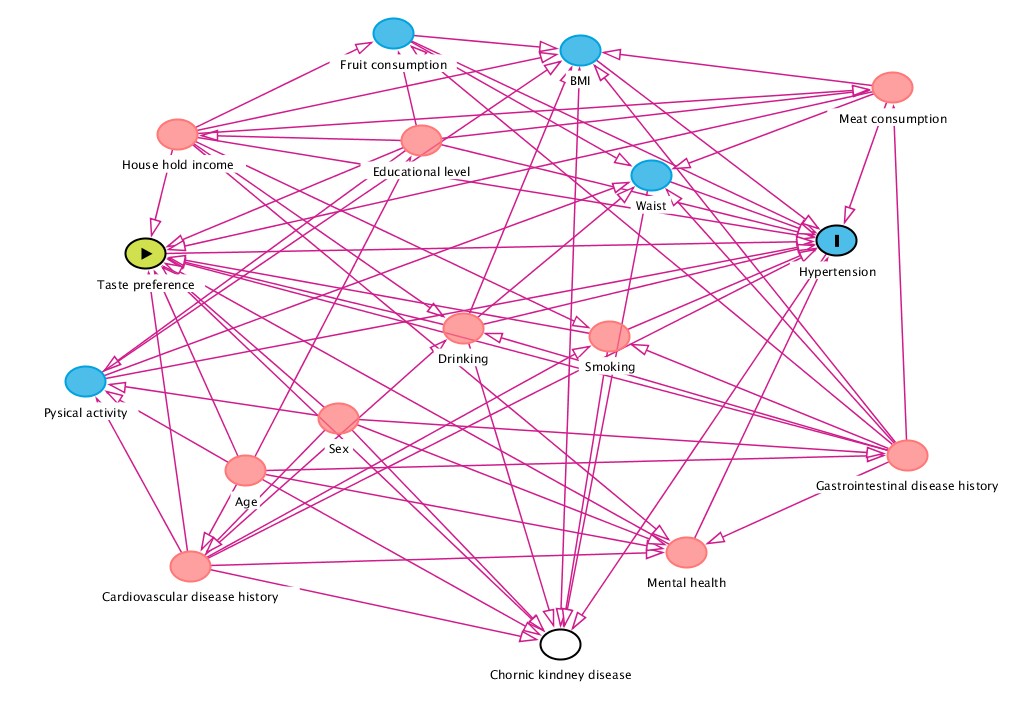


Figure S2: A directed acyclic graph (DAG) for adjusting confounders in the associations of taste preference and risk of hypertension


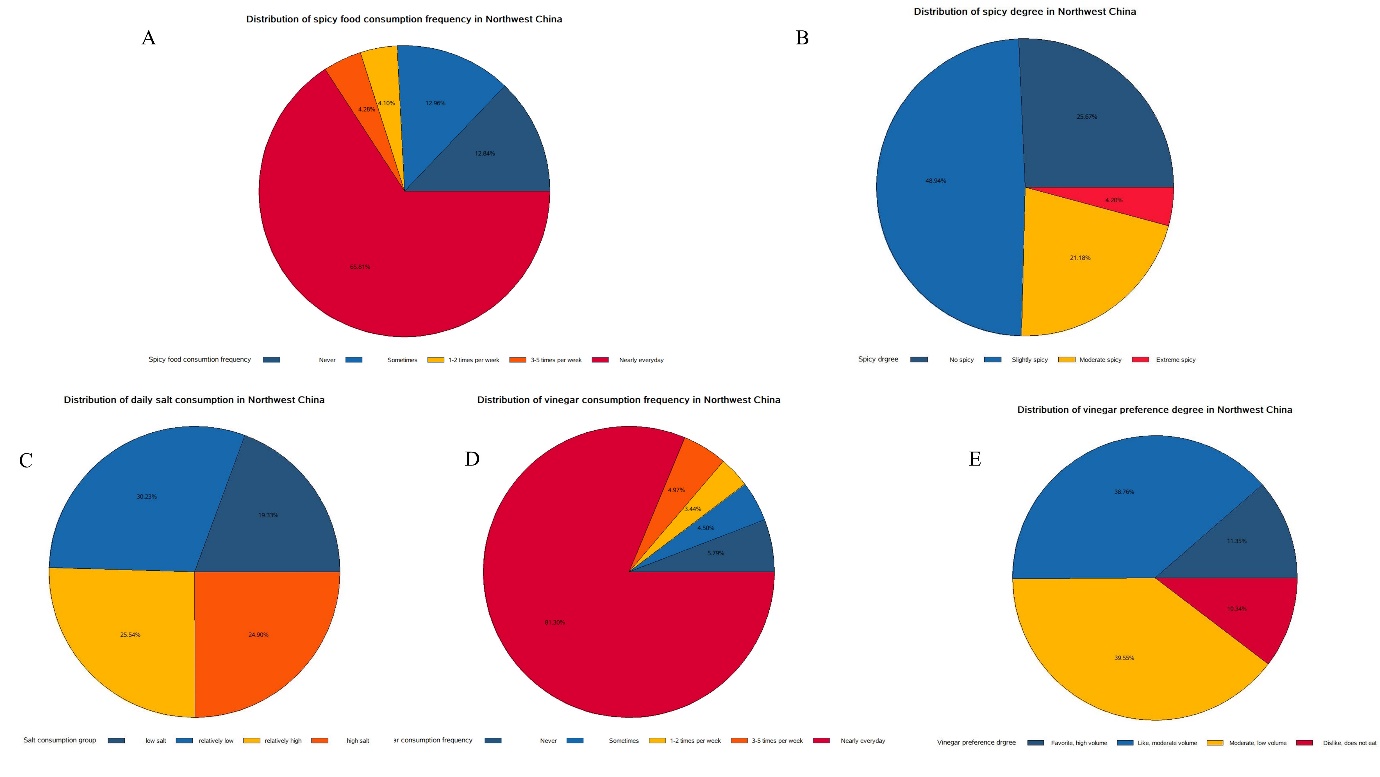


A: Distribution of spicy food consumption frequency in Northwest China

B: Distribution of spicy degrees in Northwest China

C: Distribution of daily salt consumption in Northwest China. The salt group was categorized according to the quantile of daily salt consumption. (Low salt: daily salt consumption less than 3.32g. Relatively low: daily salt consumption between 3.33g to 6.14g. Relatively high: daily salt consumption between 6.15g to 8.88g. High salt: daily salt consumption between larger than or equal to 8.89g)

D: Distribution of vinegar consumption frequency in Northwest China

E: Distribution of vinegar degree in Northwest China

Figure S3: Distribution of single taste preference in Northwest China


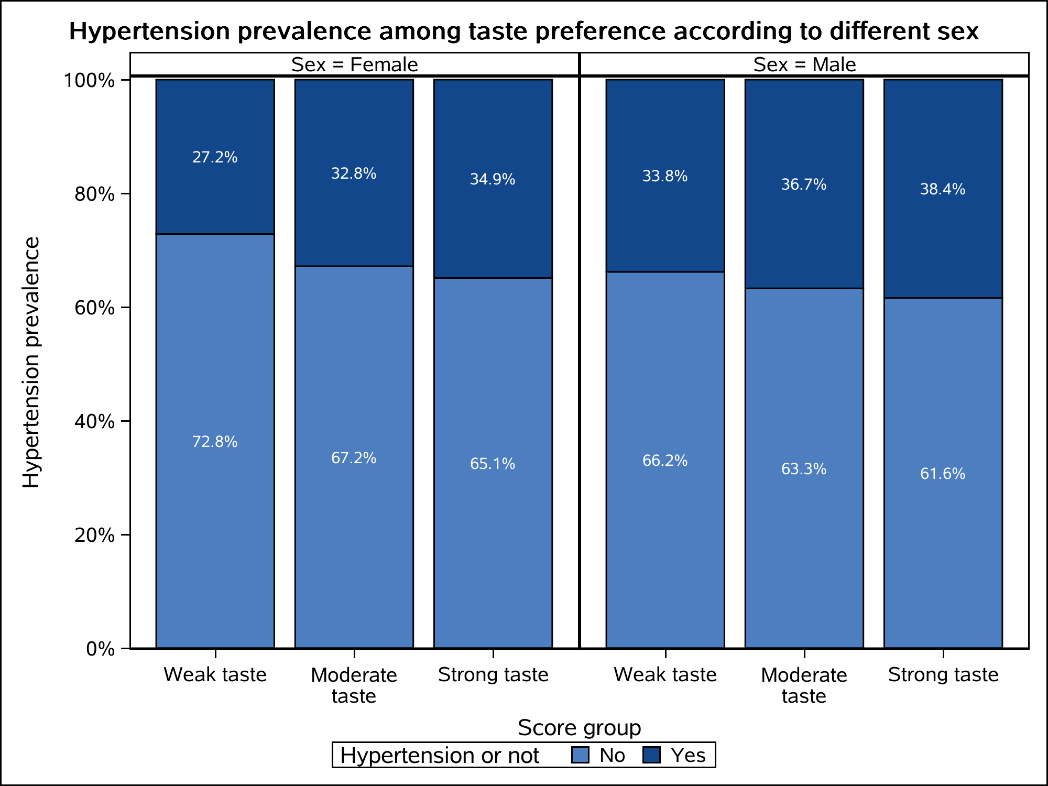


Figure S4: Hypertension prevalence among taste preferences according to different sex


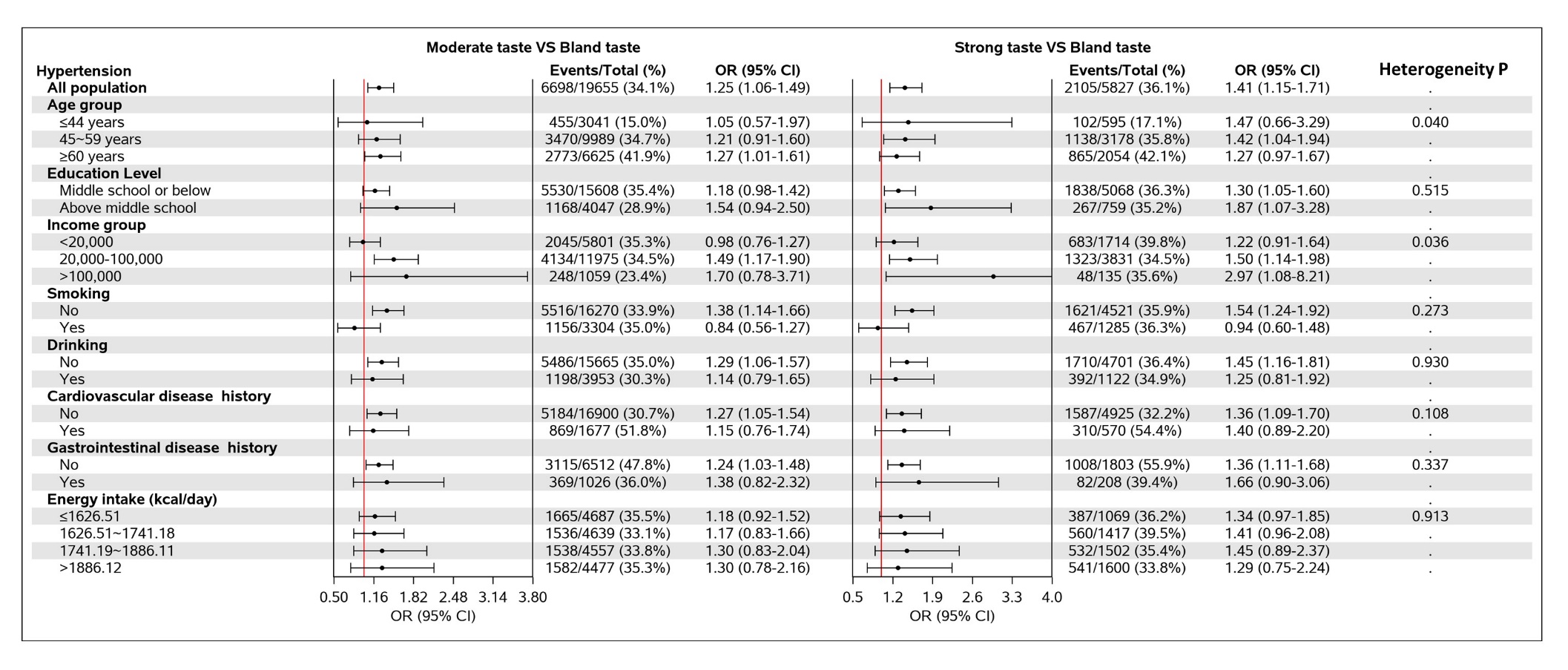
Figure S5 Forest plot of subgroup analysis for taste preference and hypertension risk

**Members of the Regional Ethnic Cohort Study collaborative group**

**Leadership:** Hong Yan (PI), Xinhua Wang (PI in Gansu Province), Jianghong Dai (PI in Xinjiang Province), Yuhong Zhang (PI in Ningxia), Xiaojie Wang (PI in Qinghai Province).

**Regional Co-ordinating Centers: School of Public Health, Xi’an Jiaotong University:** Shaonong Dang, Yuxue Bi, Lingxia Zeng, Quanli Wang, Qiang Li, Yuan Shen, Yaling Zhao, Leilei Pei, Fangyao Chen, Yijun Kang, Shengbin Xiao, Chao Li, Yue Cheng, Pengfei Qu, Baibing Mi. **Xi’an Jiaotong University Global Health Institute:** Youfa Wang. **The First Affiliated Hospital of Xi’an Jiaotong University:** Bingyin Shi, Qiumin Qu, Xinjun Lei. **The Second Affiliated Hospital of Xi’an Jiaotong University:** Zongfang Li. **Hospital of Stomatology Xi’an Jiaotong University:** Bofeng Zhu. **School of Mathematics and Statistics, Xi’an Jiaotong University:** Jian Sun, Huibin Li. **School of Management,** **Xi’an Jiaotong University:** Dehai Di. **Shaanxi Provincial CDC:** Feng Liu. **Taizhou Institute of Health Sciences, Fudan University:** Yanfeng Jiang, Tai Zhang, Xi Sun. **Baotou Medical College:** Suhua wang, Rui Qiao, Yuhang Zhao. **Gansu Provincial CDC:** Jianyun Sun, Tingcai Wang, Xiaolan Ren, Jing Zhang, Hupeng He, Lijuan Chen, Guihang Song, Shuyu Liu, Weitao Chen. **School of Public Health, Lanzhou University:** Wenlong Gao, Xiaoning Liu.

**Research Institute of Xinjiang Chinese Medicine:** Fengsen Li, Zhanjun Shu, Qi Sun. **Urumqi Municipal CDC:** Baoling Rui, Gaofeng Sun, Qin Qin. **Xinjiang Medical University:** Jinfeng Ma, Pelton Mijiti. **Changji CDC:** Jun Yang, Xiulan Kang, Jie Mi. **Ningxia CDC:** Jianhua Zhao, Yin’e Zhang, Jiancai Du, Shaoning Ma. **Ningxia Medical University:** Yi Zhao, Lan Liu, Faxuan Wang, Yajuan Zhang, Yu Zhao. **Qinghai Provincial Hospital of Cardio-cerebrovascular Diseases:** Tianyi Wu, Shiming Liu, Fengyun Liu, Deng Geng, Yuxian Li, Jialin Wen. **Qinghai Provincial CDC:** Minru Zhou, Fuchang Ma, Zhihua Xu, Xiaoping Li, Qiongyue Sha, Ji Xing, Ji Che, Qi Song, Shengjin Zhang.

**Third-Party Medical Laboratories: Shenzhen Huada Gene Research Institute:** Bo Li, Tao Li, Wei Zhang, Xiaoyun Huang, Jiayu Chen, Xinyi Shuai. **Beijing Baidu Netcom Technology Co., Ltd.:** Weijie Gan, Jian He, Qingqing Li, Wenzhi Huang, Gang Zeng.
